# Integrative Analysis of Transcriptome and Metabolome Reveals Molecular Mechanisms of Salt Tolerance in Two Citrus Rootstocks

**DOI:** 10.3390/ijms27125361

**Published:** 2026-06-14

**Authors:** Yueting Sun, Peng Wang, Yanmei Wu, Feng Liu, Longfei Jin

**Affiliations:** Institute of Citrus Research, Zhejiang Academy of Agricultural Sciences, Taizhou 318026, China; yuetingsun@126.com (Y.S.); peter_wang81@163.com (P.W.); yanm898749741@163.com (Y.W.); lfz5799@163.com (F.L.)

**Keywords:** citrus, rootstock, salt, flavonoid, transcriptome and metabolome

## Abstract

Salt stress is a major abiotic stress that threatens citrus yield and quality. To elucidate the molecular mechanisms underlying differential salt tolerance in citrus rootstocks, we performed an integrative transcriptomic and metabolomic analysis of salt-sensitive trifoliate orange (*Poncirus trifoliata*) and salt-tolerant Goutoucheng (*Citrus aurantium*) under 60 mM NaCl treatment for 12 h and 24 h. Physiological observations confirmed that Goutoucheng exhibited less growth inhibition and leaf damage than trifoliate orange. Transcriptome sequencing identified 2081 and 1588 differentially expressed genes (DEGs) in trifoliate orange at 12 h and 24 h, respectively, compared with 1166 and 997 DEGs in Goutoucheng. Metabolome profiling revealed 217 and 173 differentially accumulated metabolites (DAMs) in trifoliate orange versus 162 and 239 DAMs in Goutoucheng at the two time points. KEGG pathway analysis showed that DEGs were mainly enriched in the Mitogen-activated protein kinase (MAPK) signaling pathway—plant, plant hormone signal transduction, and flavonoid biosynthesis—and DAMs were mainly enriched in flavonoid biosynthesis, starch and sucrose metabolism, and glutathione metabolism. Integrative nine-quadrant and two-way orthogonal partial least squares analyses further pinpointed flavonoid biosynthesis as a central hub in salt response. Notably, quercetin derivatives accumulated preferentially in the salt-tolerant rootstock Goutoucheng. Several transcription factor families—including HSF, MYB, NAC, HB-HD-ZIP, C2H2, bHLH, AP2/ERF, and Trihelix—may enhance antioxidant capacity under salt stress by regulating flavonoid accumulation. Collectively, these results indicated that coordinated regulation of flavonoids contributed critically to salt stress adaptation in citrus rootstocks. The identified DEGs, DAMs, and transcription factors provide candidate targets for genetic improvement of salt tolerance in citrus.

## 1. Introduction

Excessive soil salinity is an abiotic stress that threatens the growth, development, and yield of crops around the world. Soil salinization affects about 7% of the world’s land area (950 million hectares) [1], causing $27.30 billion in economic losses annually [2]. Approximately 20–30 percent of the world’s 230 million hectares of irrigated land suffer from varying levels of soil salinization, which continues to grow due to mismanagement of fields and contamination of water for agricultural irrigation [3]. High salt content in soil can cause osmotic stress, ion toxicity, oxidative stress, and nutritional disorders in plants [4,5].

To survive salt stress, plants have developed elaborate mechanisms to perceive stress signals and accumulate salt-tolerant metabolites by modulating gene expression. The Salt Overly Sensitive (SOS) pathway plays a central role in plant salinity tolerance through ion homeostasis regulation [6]. The SOS pathway requires the combined activities of three proteins: SOS3, a calcium-binding protein, SOS2, a serine/threonine protein kinase, and SOS1, a plasma membrane-localized Na^+^/H^+^ antiporter [6,7,8]. Excessive soil salinity also caused oxidative stress on crops, leading to overproduction of reactive oxygen species (ROS) like singlet oxygen, superoxide, hydrogen peroxide, and hydroxyl radical [9]. In mango (*Mangifera indica*) plants, salt stress significantly suppressed net photosynthetic rate, stomatal conductance, and chlorophyll content, while enhancing lipid peroxidation and proline accumulation [10]. Studies on date palm (*Phoenix dactylifera*) demonstrated that biofertilizers and putrescine amine treatments effectively mitigated salt-induced oxidative stress by increasing photosynthetic pigment content, antioxidant enzyme activities including superoxide dismutase (SOD), ascorbate peroxidase (APX), and glutathione reductase (GR), and endogenous levels such as gibberellin (GA), indole-3-acetic acid (IAA), and cytokinin (CK), while decreasing the levels of lipid peroxidation and abscisic acid (ABA) accumulation [11]. Mitogen-activated protein kinases (MAPKs) are central signaling components in regulating plant growth, development, and abiotic stress tolerance [12]. Upon salt perception, MAPK cascades activate ABA signaling, which in turn modulates stomatal closure and stress-responsive gene expression [13]. MAPKs also enhance the antioxidant system by up-regulating ROS-scavenging enzymes and transcription factors [14], and they participate in the regulation of ROS metabolism [15].

Among the various secondary metabolites involved in ROS detoxification, flavonoids have attracted considerable attention due to their potent antioxidant capacity. By scavenging excess ROS, they reduce oxidative damage, thereby protecting plants from growth arrest and cell death. Numerous studies have shown that salt stress induces the biosynthesis and accumulation of flavonoids in most plants. Flavonoids served not merely as ROS scavengers but as integral modulators of hormonal, ionic, and epigenetic signaling pathways for salt acclimation [16,17,18,19]. The flavonoid biosynthetic pathway is tightly controlled by structural genes (e.g., PAL, C4H, 4CL, CHS, CHI, FLS, DFR) and regulatory transcription factors, mainly MYB, bHLH, and WD40 families. Overexpression of key enzyme genes and transcription factors in flavonoid biosynthesis significantly enhanced salt tolerance of transgenic plants [20,21,22].

Citrus is a typical glycophyte, which is sensitive to salt stress [23]. The trifoliate orange (*Poncirus trifoliata*) is the most commonly used rootstock in citrus production. The citrus grafted onto trifoliate orange has the characteristics of dwarfization, short juvenile phase, good fruit quality, and cold resistance, but is sensitive to salt stress. Goutoucheng (*Citrus aurantium*) is an original local citrus rootstock resource in Zhejiang province, which was widely used in coastal citrus orchards. The citrus grafted in Goutoucheng grew fast and has more roots and a strong tolerance to salt stress. Despite this known difference in salt tolerance, the molecular mechanisms underlying the early response to salt stress—particularly the key genes, metabolites, and regulatory pathways—remain largely unknown in these two rootstocks. Therefore, the specific objectives of this study were: (1) to characterize the physiological responses of the two rootstocks under 60 mM NaCl treatment; (2) to identify differentially expressed genes (DEGs) and differentially accumulated metabolites (DAMs) 12 h and 24 h after salt treatment using transcriptomic and metabolomic profiling; (3) to determine the key metabolic pathways, especially flavonoid biosynthesis, that distinguish the salt-tolerant Goutoucheng from the salt-sensitive trifoliate orange.

In this study, transcriptome and metabolome were used to analyze the gene expression and metabolic accumulation of two citrus rootstocks with significant differences in salt tolerance. The key genes and metabolites of citrus response were excavated, and the regulatory network of salt tolerance in citrus was constructed. The results of this study will be helpful in understanding the molecular mechanism of salt tolerance in citrus, as well as provide a theoretical basis for genetic improvement of salt tolerance in citrus rootstocks and development of new technology for cultivation.

## 2. Results

### 2.1. Physiological Response of Two Citrus Rootstocks to Salt Stress

**Figure 1:**
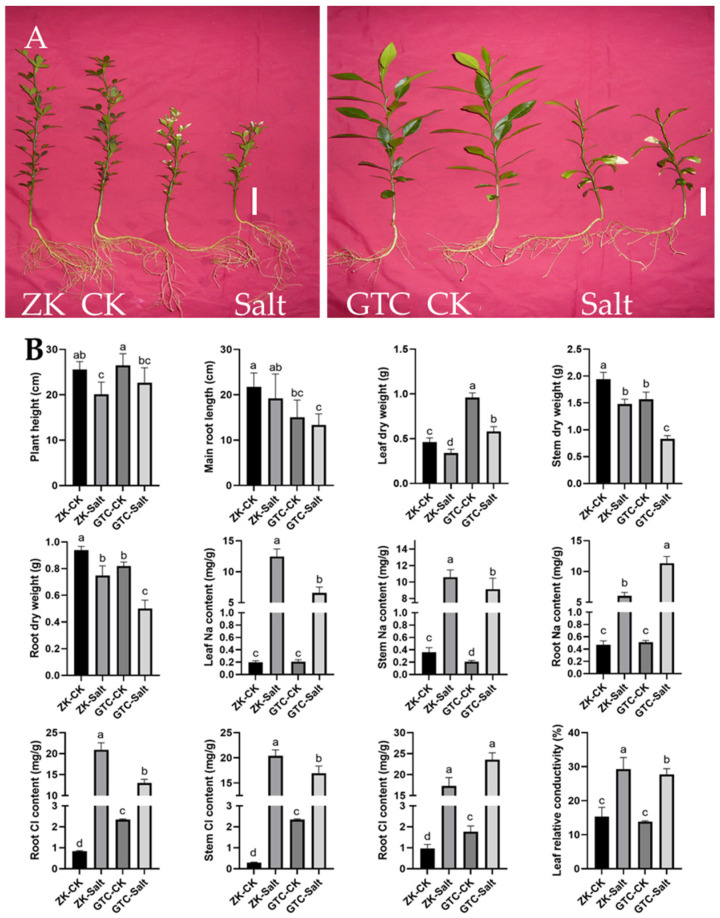
Effect of salt treatment on citrus growth. (**A**) Phenotype of ZK and GTC under control and salt-treatment conditions. ZK refers to trifoliate orange (*Poncirus trifoliata*), and GTC refers to Goutoucheng (*Citrus aurantium*). Scale bar = 5 cm. (**B**) Growth parameters and physiological indexes of two citrus rootstocks under salt treatment. The values are presented as mean ± SD. The different lowercase letters indicate significant differences at *p* < 0.05.

Salt treatment induced visible phenotypic changes in both rootstocks. Trifoliate orange (ZK) exhibited more severe growth inhibition and leaf damage compared to Goutoucheng (GTC) (Figure 1A). Physiological parameter measurements, including plant height, root length, biomass, and antioxidant enzyme activities, showed significant differences between the two rootstocks under salt stress (Figure 1B), confirming the superior salt tolerance of GTC.

### 2.2. Transcriptomic Profiling Under Salt Stress

**Figure 2:**
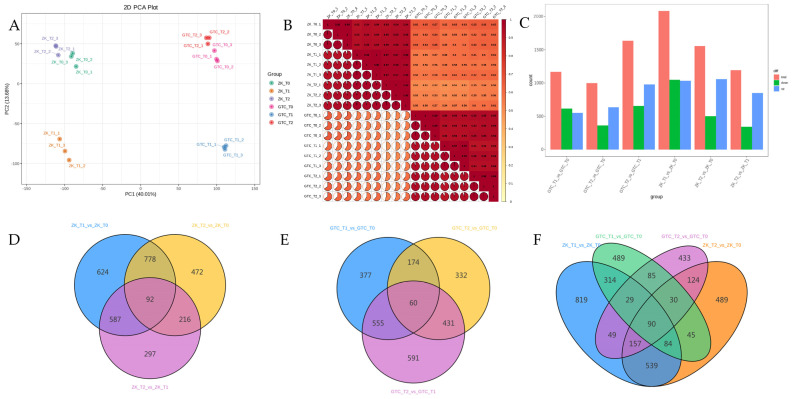
Overview of transcriptomics response of two citrus rootstocks to salt stress. (**A**) Principal component analysis (PCA) plots of transcripts identified by RNA-seq of salt-stressed citrus roots at 0, 12, and 24 h after stress. (**B**) Correlation analysis diagram of samples. (**C**) Statistics of DEGs. (**D**) Venn diagram of DEGs of trifoliate orange. (**E**) Venn diagram of DEGs of Goutoucheng. (**F**) Venn diagram of DEGs of two citrus rootstocks.

After removing the adapters and low-quality reads, RNA-seq analysis of 18 libraries generated 159.82 GB of clean reads (Table S1). Transcriptomic quality control analysis showed that the error rate, Q20 values, Q30 values, GC content, and read distribution met the requirements of transcriptome sequencing (Table S1). The mapping analysis of clear reads to the genome showed that 75.44–89.47% of reads were mapped to the reference genome (Table S2). PCA result showed that the PC1 explained 13.68% of the variation in salt treatment, while the PC2 explained 40.01% of the variation (Figure 2A). Correlation analysis showed that the correlation coefficient between biological replicates was as high as 95% (Figure 2B). As shown in Figure S1, the expression patterns of all 6 genes measured by qRT-PCR were highly consistent with those from RNA-seq, indicating the high reliability of our transcriptomic data. DEGs were screened according to the threshold of fold change ≥ 2 and FDR ≤ 0.05. After 12 h of salt stress treatment, 2081 genes were differentially expressed (1032 up-regulated and 1049 down-regulated) in trifoliate orange, and 1166 genes were differentially expressed (551 up-regulated and 615 down-regulated) in Goutoucheng. After treatment for 24 h, 1558 genes were differentially expressed (1056 up-regulated, 502 down-regulated) in trifoliate orange, and 997 genes were differentially expressed (636 up-regulated, 361 down-regulated) in Goutoucheng (Figure 2C). Venn diagram analysis of trifoliate orange under salt treatment showed that 1481 genes were differently expressed at 12 h, 688 genes were differently expressed at 12 h, and 92 genes were differently expressed at both 12 h and 24 h (Figure 2D). Venn diagram analysis of Goutoucheng under salt treatment showed that 943 genes were differently expressed at 12 h, 763 genes were differently expressed at 12 h, and 60 genes were differently expressed at both 12 h and 24 h (Figure 2E). Venn diagram analysis of the differential expression of the two citrus rootstocks under salt stress at different periods showed that 60 genes were common in the varieties and treatments (Figure 2F).

### 2.3. Enrichment Analysis of Differentially Expressed Genes in Two Citrus Rootstocks Under Salt Stress

**Figure 3:**
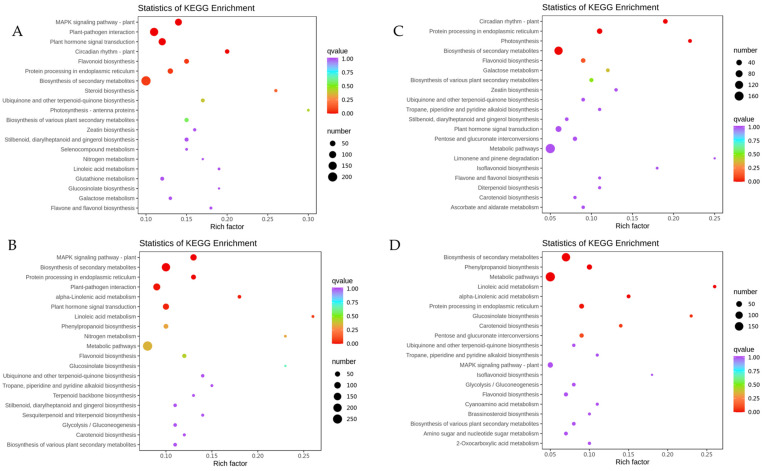
KEGG pathway analysis of differential expression genes of trifoliate orange at 0 h vs. 12 h (**A**), 0 h vs. 24 h (**B**), and Goutoucheng at 0 h vs. 12 h (**C**), 0 h vs. 24 h (**D**) after salt treatment.

In order to study the function of candidate genes of citrus rootstocks in response to salt stress, the KEGG database was used to conduct enrichment analysis of DEGs. A large number of differentially expressed genes were enriched in the MAPK signaling pathway—plant, plant hormone signal transduction, flavonoid biosynthesis, biosynthesis of secondary metabolites, protein processing in the endoplasmic reticulum (Figure 3). The DEGs of trifoliate orange at 12 h after salt treatment were mainly enriched in the MAPK signaling pathway—plant, plant–pathogen interaction, plant hormone signal transduction, circadian rhythm, plant, and flavonoid biosynthesis (Figure 3A). The DEGs of trifoliate orange at 24 h after salt treatment were mainly enriched in the MAPK signaling pathway—plant, biosynthesis of secondary metabolites, protein processing in endoplasmic reticulum, plant–pathogen interaction, and alpha-linolenic acid metabolism (Figure 3B). The DEGs of Goutoucheng at 12 h after salt treatment were mainly enriched in circadian rhythm—plant, protein processing in endoplasmic reticulum, photosynthesis, biosynthesis of secondary metabolites, and flavonoid biosynthesis (Figure 3C). The DEGs of Goutoucheng at 24 h after salt treatment were mainly enriched in the biosynthesis of secondary metabolites, phenylpropanoid biosynthesis, metabolic pathways, linoleic acid metabolism, and alpha-linolenic acid metabolism (Figure 3D).

### 2.4. Metabolomics Analysis of the Response of Two Citrus Rootstocks to Salt Stress

**Figure 4:**
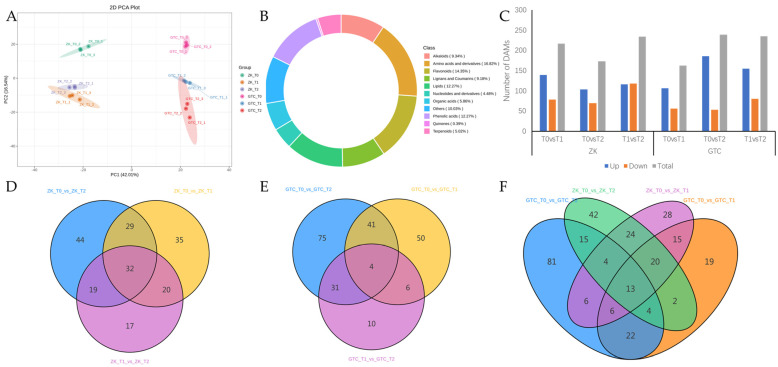
Overview of metabolomics response of two citrus rootstocks to salt stress. (**A**) Principal component analysis (PCA) of all samples. PC1 and PC2 explained 42.01% and 16.54% of the total variance, respectively. (**B**) Correlation heatmap showing high reproducibility among biological replicates (r > 0.95). (**C**) Number of DAMs in trifoliate orange (ZK) and Goutoucheng (GTC) at 12 h (T1) and 24 h (T2) after salt treatment. (**D**–**F**) Venn diagrams showing DAM overlaps in ZK (**D**), GTC (**E**), and between the two rootstocks (**F**).

Metabolomics quality control analysis showed that the coefficient of variation met the requirements of metabolomics sequencing (Figure 4A,B). PCA results showed that there was a high degree of separation among different salt treatments and rootstock varieties (Figure 4A). The R^2^-value greater than 0.95 between biological replicates implied that the reproducibility of metabolomic data was reliable (Figure 4B). A total of 1296 metabolites including 218 amino acids and derivatives (16.82%), 159 phenolic acids (12.27%), 58 nucleotides and derivatives (4.48%), 186 flavonoids (14.35%), 119 lignans and coumarins (9.18%), 121 alkaloids (9.34%), 65 terpenoids (5.02%), 76 organic acids (5.86%), 159 lipids (12.27), quinones (0.39%), and 130 others (10.03%) were detected based on UPLC-MS/MS in 18 samples (Figure 4B). As shown in Figure 4C, 139 up-regulated and 78 down-regulated, 103 up-regulated and 70 down-regulated, and 116 up-regulated and 118 down-regulated metabolites were identified in T0 vs. T1, T0 vs. T2, and T1 vs. T2 of trifoliate orange; 106 up-regulated and 56 down-regulated, 186 up-regulated and 53 down-regulated, and 155 up-regulated and 80 down-regulated metabolites were identified in T0 vs. T1, T0 vs. T2, and T1 vs. T2 of GTC. Venn diagram analysis of trifoliate orange under salt treatment showed that 63 metabolites were differently expressed at 12 h, 55 metabolites were differently expressed at 12 h, and 61 metabolites were differently expressed at both 12 h and 24 h (Figure 4D). Venn diagram analysis of GTC under salt treatment showed that 106 metabolites were differently expressed at 12 h, 55 metabolites were differently expressed at 12 h, and 61 metabolites were differently expressed at both 12 h and 24 h (Figure 4E). Venn diagram analysis of the differential expression of the two citrus rootstocks under salt stress at different periods showed that 60 genes were similar in varieties and treatments (Figure 4F).

### 2.5. Enrichment Analysis of DAMs in Two Citrus Rootstocks Under Salt Stress

**Figure 5:**
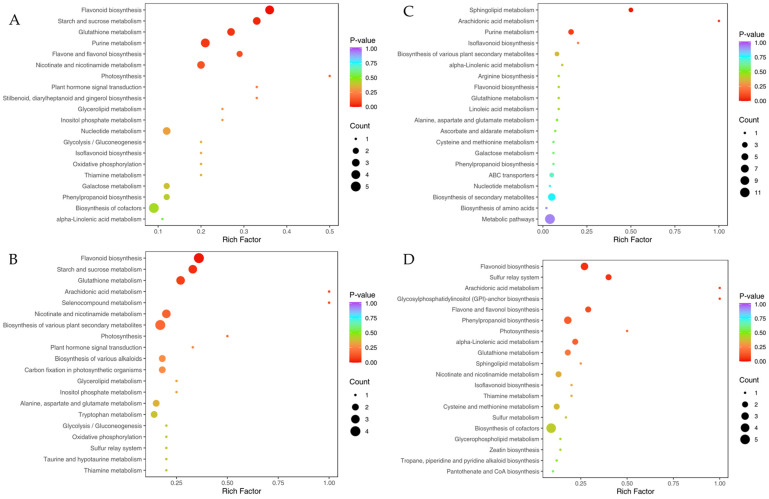
KEGG pathway analysis of DAMs in two citrus rootstocks under salt stress. (**A**) Trifoliate orange at 12 h vs. 0 h; (**B**) trifoliate orange at 24 h vs. 0 h; (**C**) Goutoucheng at 12 h vs. 0 h; (**D**) Goutoucheng at 24 h vs. 0 h.

In order to study the function of candidate metabolites in response to citrus salt stress, the KEGG database was used to conduct enrichment analysis of DAMs. The DAMs of trifoliate orange at 12 h after salt treatment were mainly enriched in flavonoid biosynthesis, starch and sucrose metabolism, glutathione metabolism, purine metabolism, and flavone and flavonol biosynthesis (Figure 5A). The DAMs of trifoliate orange at 24 h after salt treatment were mainly enriched in flavonoid biosynthesis, starch and sucrose metabolism, glutathione metabolism, arachidonic acid metabolism, and selenocompound metabolism (Figure 5B). The DAMs of Goutoucheng at 12 h after salt treatment were mainly enriched in sphingolipid metabolism, arachidonic acid metabolism, purine metabolism, isoflavonoid biosynthesis, and biosynthesis of various plant secondary metabolites (Figure 5C). The DAMs of Goutoucheng at 24 h after salt treatment were mainly enriched in flavonoid biosynthesis, sulfur relay system, arachidonic acid metabolism, glycosylphosphatidylinositol (GPI)-anchor biosynthesis, and flavone and flavonol biosynthesis (Figure 5D).

### 2.6. Transcriptome and Metabolome Association Analysis of Two Citrus Rootstocks Under Salt Stress

To gain deeper insights into the regulatory relationships between gene expression and metabolite accumulation, we performed nine-quadrant association analysis and O2PLS analysis on the transcriptomic and metabolomic data.

**Figure 6:**
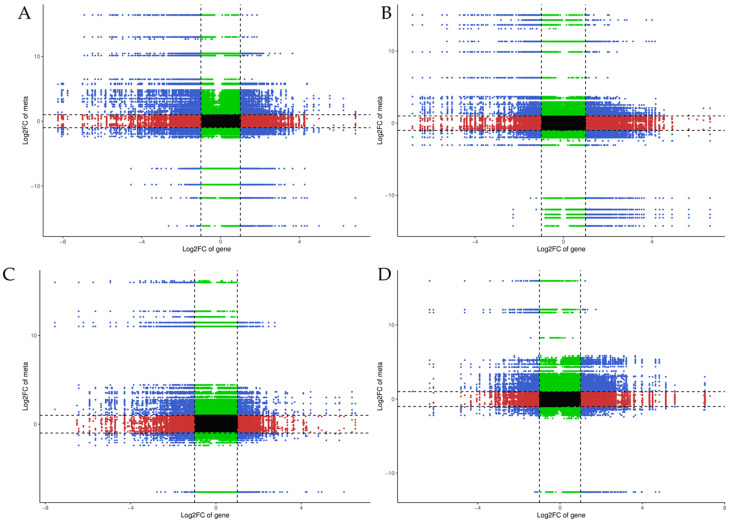
Nine-quadrant analysis of the correlation between DEGs and DAMs in trifoliate orange at 0 h vs. 12 h (**A**), 0 h vs. 24 h (**B**), and Goutoucheng at 0 h vs. 12 h (**C**), 0 h vs. 24 h (**D**) after salt treatment. Note: The black dotted line is divided into 1 to 9 quadrants from left to right and from top to bottom in sequence.

Nine-quadrant analysis categorized genes and metabolites into nine regions based on their expression/accumulation patterns, clearly illustrating their coordinated changes under salt stress (Figure 6). The analysis revealed that in both rootstocks, there existed a set of gene–metabolite pairs where up-regulated genes corresponded to increased metabolite accumulation (or down-regulated genes corresponded to decreased accumulation). These pairs were significantly enriched in pathways such as flavonoid biosynthesis, starch and sucrose metabolism, and glutathione metabolism. This statistically confirmed the activity of these metabolic pathways during salt stress response and their direct regulation at the transcriptional level.

**Figure 7:**
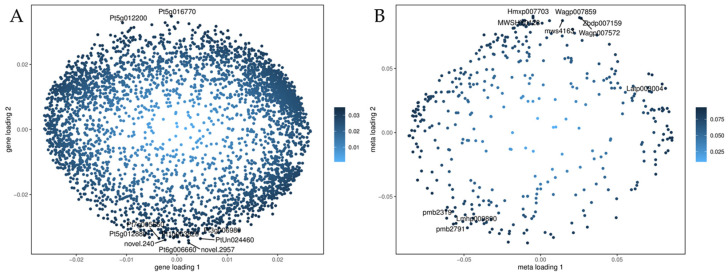
O2PLS analysis on genes (**A**) and metabolites (**B**) loading sort of two citrus rootstocks under salt stress.

Further O2PLS analysis (Figure 7) was employed to identify key variables driving phenotypic changes. Loading sort plots indicated that the genes contributing most to distinguishing salt stress responses included Calmodulin (Pt5g016770), Cytochrome P450 (Pt5g012200), HSF30 (Pt1g003820), hypothetical protein (novel.240), C4H (PtUn024460), HSP70 (Pt7g005660), *TCP21* (Pt3g006980), and three unknown genes (novel.2957, novel.240, Pt5g012880). Concurrently, the metabolites with the highest contribution scores were primarily flavonoids (e.g., quercetin 3,5,7,3′-tetramethyl ether), lipids (e.g., 9-Hydroperoxy-10E,12,15Z-octadecatrienoic acid), and two sugars including Nystose (mws4163) and 2-Methyl-5-acetonyl-7-hydroxychromone glucoside (Latp003004). This result suggested that under salt stress, citrus rootstocks remodel the metabolic flux of flavonoids, lipids, and sugars by regulating the expression of specific genes. These metabolic alterations constituted the core biochemical basis for their adaptation to saline environments.

## 3. Discussion

Citrus is an essential horticultural fruit with a strong flavor and good taste. However, soil salinity is a serious threat to the yield and quality of citrus, especially to the orange orchard of the sea coating and facility greenhouse [24]. Identification of key genes and metabolites of citrus response to salt stress may reveal the regulatory mechanism, which could provide a theoretical basis for the salt-tolerant molecular orientation breeding and development of salt-tolerant cultivation technology. In this study, two citrus rootstocks were used for transcriptomic and metabolomic integrative analysis to discover the mechanism of short-term salt response in citrus.

### 3.1. MAPK Signaling and Its Link to Flavonoid Metabolism

MAPK cascades are an important pathway for plants to sense and cascade external signals, which play a key role in the regulation of plant growth and development and biological biotic and abiotic stress responses [25]. In the study of rice (*Oryza sativa* L.), 24 DEGs related to the MAPK cascade pathway were identified in shoots and roots under salt stress [26]. Six MAPK genes were induced during salinity treatment in sweet orange (*Citrus sinensis*) [27]. In *Arabidopsis*, activation of MAPK kinase 9 enhanced sensitivity to salt stress through inducing ethylene and camalexin biosynthesis, whereas loss of activity of *mkk9* mutant reduced salt sensitivity [28]. Overexpression of *PeMKK2a* increased salt tolerance of transgenic poplar (*Populus trichocarpa*) via improving activities of SOD, CAT, and POD [14]. Knockout mutant of *AtMKK1* elevated salt tolerance during both germination and adulthood [29]. In this study, after salt stress, a large number of differentially expressed genes were significantly enriched in MAPK signaling pathways in the root tips of two citrus rootstocks. Two *MAPK* genes (Pt2g028590, Pt7g017310) involved in salt and osmotic stress adaptation were up-regulated in trifoliate orange.

Flavonoids are the key secondary metabolites in crop growth and development, quality formation, and biotic and abiotic stress response [30]. For example, salt stress significantly increased the content of total flavonoids in the sprouted seeds of bean (*Phaseolus vulgaris*), the leaves and suspended cells of *Ginkgo biloba*, the leaves of *Ligustrum* spp. and willow (*Salix* spp.), and the roots of *Sorghum bicolor* [18,31]. Omics studies have shown that a large number of differentially expressed genes are enriched in flavonoid biosynthesis pathways under salt stress [18,32]. Importantly, emerging evidence indicates that MAPK cascades can modulate flavonoid biosynthesis by phosphorylating transcription factors or regulating the expression of key biosynthetic genes [33]. In this study, 48 and 35 DEGs related to flavonoid biosynthesis were identified in the roots of trifoliate orange and Goutoucheng under salt stress. Thus, the activation of MAPK pathways observed in this study may contribute to salt tolerance not only through antioxidant enzyme activation but also by promoting flavonoid accumulation. However, it should be noted that our data are derived from transcriptomic and metabolomic correlation analyses and do not provide direct functional validation. Whether flavonoid accumulation causally contributes to salt tolerance in citrus rootstocks requires further investigation using overexpression or silencing of key flavonoid biosynthetic genes.

### 3.2. Key DAMs of Citrus Rootstock Response to Salt Stress

Flavonoids are a group of polyphenol substances including flavonols, flavones, isoflavones, anthocyanidins, flavanones, flavanols, and chalcones [31], which are widely distributed in citrus plants [34]. As non-enzymatic antioxidants, flavonoids directly scavenge excessive reactive oxygen species, including hydroxyl radicals, hydrogen peroxide, and superoxide anions, by donating hydrogen atoms or electrons, thereby mitigating oxidative harm to DNA, proteins, and membrane lipids [35,36]. In addition, the dihydroxy forms of flavonoids chelate metal ions such as ferrous and cuprous ions, thereby limiting their participation in the Fenton reaction and suppressing the generation of hydroxyl radicals [37]. Furthermore, flavonoids enhance the expression of antioxidant-related genes and up-regulate the activity of enzymatic antioxidants, including peroxidase and glutathione reductase, through the activation of transcription factors such as MYB [38]. This establishes a synergistic antioxidant network that combines enzymatic and non-enzymatic components [35,36].

In this study, flavonoids were the most abundant class of DAMs in the two citrus rootstocks under salt stress. In trifoliate orange, after 12 h of salt treatment, 2 flavonoids were up-regulated and 21 down-regulated; at 24 h, 19 were up-regulated and 7 down-regulated. In GTC, 5 flavonoids were up-regulated and 16 down-regulated at 12 h, while 16 were up-regulated at 24 h. These dynamic changes suggested that flavonoid metabolism was temporally regulated in response to salt stress in citrus. Specific flavonoids, such as quercetin derivatives, were found to accumulate preferentially in the salt-tolerant rootstock (GTC). Quercetin has been shown to enhance salt tolerance in tomato by improving antioxidant defense and glyoxalase systems [39]. Similarly, the accumulation of flavonols and flavones in citrus rootstocks may contribute to reactive oxygen species (ROS) scavenging, thereby alleviating oxidative stress under salt stress [33,40]. The enrichment of DAMs in flavonoid biosynthesis pathways, as revealed by KEGG analysis, further supports the central role of flavonoids in citrus salt tolerance.

### 3.3. Transcription Factors Involved in Flavonoid Metabolism of Citrus Rootstocks Response to Salt Stress

Transcription factors play a critical role in orchestrating flavonoid biosynthesis under stress conditions [41,42,43,44,45]. In this study, the expression of *HSF* (Pt3g031640), *MYB* (Pt3g035480, Pt9g007770), *NAC* (Pt4g000070), *WRKY* (Pt4g012940), *HB-HD-ZIP* (Pt6g009450), *C2H2* (Pt6g015990), *bHLH* (Pt1g009430), *AP2/ERF* (PtUn031000) and Trihelix (Pt6g006520) were significantly induced by salt stress in both trifoliate orange and Goutoucheng rootstocks. Among these, MYB and bHLH TFs are well-known regulators of flavonoid biosynthesis, directly binding to promoters of structural genes such as CHS, CHI, and FLS [46]. For instance, overexpression of *AtMYB12* in tomato (*Solanum lycopersicum*) substantially increased flavonoid content and enhanced stress tolerance [22]. In apple (*Malus domestica*), *MdMYB9* and *MdMYB11* positively regulated flavonoid content through directly binding to the promoter elements of *ANS*, *UFGT*, *ANR*, and *DFR,* with MdbHLH3 acting as a co-regulator [46]. Although our data do not provide functional validation, the coordinated expression of these TFs with flavonoid biosynthetic genes demonstrated that HSF, MYB, NAC, HB-HD-ZIP, C2H2, bHLH, AP2/ERF, and Trihelix may enhance the antioxidant capacity under salt stress by regulating the accumulation of flavonoids in citrus rootstocks.

## 4. Materials and Methods

### 4.1. Plant Growth and Salt Treatment

Fifteen-week-old seedlings of trifoliate orange (*Poncirus trifoliata*) and Goutoucheng (*Citrus aurantium*) seedlings were grown in greenhouse at the Zhejiang Citrus Research Institute, Taizhou, China (28°38′ N, 121°9′ E). The seedlings were irrigated with Hoagland nutrient solution (pH 6.0), which was aerated for 20 min every 2 h and renewed twice a week.

For salt stress treatment, 60 mM NaCl was added to the nutrient solution. Root tip samples were collected at 0, 12, and 24 h after treatment. For each treatment and time point, six seedlings were pooled to form one biological replicate, with three biological replicates per condition. The samples were named as follows: ZK_T0, ZK_T1, ZK_T2 for trifoliate orange (0, 12, 24 h) and GTC_T0, GTC_T1, GTC_T2 for Goutoucheng. All root samples were immediately frozen in liquid nitrogen and stored at −80 °C until further analysis.

### 4.2. RNA Extraction and Transcriptome Analysis

Total RNA was extracted from approximately 100 mg of frozen root tissue using TRIzol reagent (Invitrogen, Carlsbad, CA, USA). RNA quantity and purity of all samples were analyzed using Bioanalyzer 2100 and RNA 6000 Nano LabChip Kit (Agilent, Santa Clara, CA, USA). A total of 18 cDNA libraries were constructed using NEBNext^®^UltraTM RNA Library Prep Kit for Illumina^®^ (NEB, Ipswich, MA, USA). Sequencing was performed on an Illumina^®^ HiSeq2500 platform (Illumina, San Diego, CA, USA). Each sample was sequenced in triplicate. The raw reads were filtered by removing low-quality reads and adapters. The clean reads were mapped to the *Poncirus trifoliata* genome [47] using HISAT v2.1.0. New genes were predicted using StringTie v1.3.4d. Gene expression levels were quantified as fragments per kilobase of transcript per million mapped reads (FPKM) using featureCounts v1.6.2. DEGs were screened by DESeq2 v1.22.1 through fold change (FC) ≥ 2 and a false discovery rate (FDR) < 0.05. DEGs were then subjected to enrichment analysis of GO functions and KEGG pathways. The transcriptome sequencing was performed by Metware Biotechnology Co., Ltd. (Wuhan, China).

### 4.3. Metabolite Extraction and Metabolome Analysis

The frozen root samples were freeze-dried using a lyophilizer (Scientz-100F, Ningbo, China) and ground into powder form using a grinder (MM 400, Retsch, Haan, Germany). Next, 50 mg of sample powders were extracted by adding 1200 μL of −20 °C pre-cooled 70% methanolic and internal standard. After vortexing and centrifugation, the supernatant was aspirated and filtered through a 0.22 μm microporous membrane and stored in an injection vial for UPLC-MS/MS analysis. The sample extracts were analyzed using a UPLC and MS/MS system (UPLC, ExionLC™ AD, SCIEX, Framingham, MA, USA; MS/MS, Applied Biosystems 6500 Q TRAP, SCIEX, Framingham, MA, USA) by the Metware Biotechnology Co., Ltd. (Wuhan, China). The qualitative analysis of primary and secondary metabolite was performed by searching the UPLC-MS/MS detection platform and the self-built database MWDB. Metabolites were identified by comparing the *m*/*z* values, RT, and fragmentation patterns with the standards. Principal component analysis (PCA) and hierarchical cluster analysis (HCA) were performed using R software v4.4.0. DAMs were screened using the R package MetaboAnalystR through VIP > 1 and |Log2FC| ≥ 1.0. Identified metabolites were annotated using the KEGG Compound database (http://www.kegg.jp/kegg/compound/, accessed on 10 October 2024). Annotated metabolites were then mapped to the KEGG Pathway database (http://www.kegg.jp/kegg/pathway.html, accessed on 20 October 2024). Pathways with significantly regulated metabolites mapped were then fed into metabolite sets enrichment analysis (MSEA), their significance determined by hypergeometric test’s *p*-values.

### 4.4. Integrated Metabolome and Transcriptome Analyses

Pearson correlation coefficients (r) between the metabolome and transcriptome data were calculated. r > 0.8 was selected for further analysis. To better understand the relationship between genes and metabolites, the results of DAM analysis were combined with the results of DEGs analysis, and the comparison group of DAMs and DEGs was mapped to the KEGG pathway diagram at the same time. Nine-quadrant and O2PLS analyses were performed to identify coordinated changes between gene expression and metabolite accumulation. Cytoscape (version 2.8.2) was used to visualize the relationship between the metabolome and the transcriptome.

### 4.5. Quantitative Real-Time PCR Analysis

RT-qPCR was used to verify the RNA-seq results. A total of 6 genes were randomly selected from DEGs. Primers were designed using NCBI Primer-BLAST website (https://www.ncbi.nlm.nih.gov/tools/primer-blast/, accessed on 29 April 2026). The primer sequences were listed in Table S3. RT-qPCR was performed using TB Green^®^ Fast qPCR Mix (Takara, Kyoto, Japan) according to the manufacturer’s instructions. All RT-qPCR analyses were performed using three technical and three biological replicates. The citrus *Actin* gene was used as an internal control to check the expression. The relative expression levels were calculated by the 2^−ΔCT^ method.

### 4.6. Statistical Analysis

All experiments consisted of three replicates (n = 3). Student’s *t*-test was used for two-group comparisons, whereas one-way ANOVA followed by Tukey’s range test were used for multiple comparisons. Data are expressed as the mean ± standard deviation (SD) of the three replicates. The SPSS software (version 20.0; SPSS, Chicago, IL, USA) was used for statistical analysis. Results were considered statistically significant at *p* < 0.05.

## 5. Conclusions

In this study, an integrated transcriptomic and metabolomic analysis revealed the molecular mechanisms underlying salt stress responses in two citrus rootstocks. Flavonoid biosynthesis is a core pathway in citrus rootstocks under salt stress, as it was significantly enriched at both the transcriptional and metabolic levels and contributes to salt tolerance by scavenging reactive oxygen species. Multiple transcription factors (HSF, MYB, NAC, bHLH, AP2/ERF, etc.) are involved in regulating flavonoid metabolism, synergistically enhancing antioxidant defense capacity. Goutoucheng exhibited a more sustained metabolic adaptation during the later stage of salt stress, with stronger co-regulation in glutathione and carbohydrate metabolism pathways compared with trifoliate orange, correlating with its superior salt tolerance. Key regulatory genes (e.g., HSF30, C4H) and key metabolites (flavonoids) were identified, providing candidate targets for genetic improvement of salt tolerance in citrus rootstocks. This study provides a theoretical foundation for understanding salt stress responses in citrus and for breeding salt-tolerant rootstock varieties.

## Data Availability

Raw reads of RNA-seq have been uploaded in the GSA (Genome Sequence Archive) database (BioProject accession number was PRJCA026576).

## Supplementary Materials

The following supporting information can be downloaded at: https://www.mdpi.com/article/10.3390/ijms27125361/s1.

## References

[1] Liang X., Li J., Yang Y., Jiang C., Guo Y. (2024). Designing salt stress-resilient crops: Current progress and future challenges. J. Integr. Plant Biol..

[2] Morton M.J.L., Awlia M., Al-Tamimi N., Saade S., Pailles Y., Negrão S., Tester M. (2019). Salt stress under the scalpel-dissecting the genetics of salt tolerance. Plant J..

[3] Ismail A.M., Horie T. (2017). Genomics, physiology, and molecular breeding approaches for improving salt tolerance. Annu. Rev. Plant Biol..

[4] Yang Y., Guo Y. (2018). Elucidating the molecular mechanisms mediating plant salt-stress responses. New Phytol..

[5] Zhang H., Zhu J., Gong Z., Zhu J. (2022). Abiotic stress responses in plants. Nat. Rev. Genet..

[6] Ali A., Petrov V., Yun D., Gechev T. (2023). Revisiting plant salt tolerance: Novel components of the SOS pathway. Trends Plant Sci..

[7] Wu S., Ding L., Zhu J. (1996). SOS1, a Genetic locus essential for salt tolerance and potassium acquisition. Plant Cell.

[8] Dai W., Wang M., Gong X., Liu J.H. (2018). The transcription factor *FcWRKY40* of *Fortunella crassifolia* functions positively in salt tolerance through modulation of ion homeostasis and proline biosynthesis by directly regulating SOS2 and P5CS1 homologs. New Phytol..

[9] Yang Y., Guo Y. (2018). Unraveling salt stress signaling in plants. J. Integr. Plant Biol..

[10] Elsheery N., Helaly M., El-Hoseiny H., Alam-Eldein S. (2020). Zinc oxide and silicone nanoparticles to improve the resistance mechanism and annual productivity of salt-stressed mango trees. Agronomy.

[11] Naser H., Hanan E., Elsheery N., Kalaji H. (2016). Effect of biofertilizers and putrescine amine on the physiological features and productivity of date palm (*Phoenix dactylifera*, L.) grown on reclaimed-salinized soil. Trees.

[12] Chen X., Ding Y., Yang Y., Song C., Wang B., Yang S., Guo Y., Gong Z. (2021). Protein kinases in plant responses to drought, salt, and cold stress. J. Integr. Plant Biol..

[13] de Zelicourt A., Colcombet J., Hirt H. (2016). The role of MAPK modules and ABA during abiotic stress signaling. Trends Plant Sci..

[14] Wang J., Sun Z., Chen C., Xu M. (2022). The MKK2a Gene Involved in the MAPK signaling cascades enhances *Populus* salt tolerance. Int. J. Mol. Sci..

[15] Wei L., Feng L., Liu Y., Liao W. (2022). Mitogen-activated protein kinase is involved in salt stress response in tomato (*Solanum lycopersicum*) seedlings. Int. J. Mol. Sci..

[16] Zhang L., Zhang Z., Fang S., Liu Y., Shang X. (2021). Integrative analysis of metabolome and transcriptome reveals molecular regulatory mechanism of flavonoid biosynthesis in *Cyclocarya paliurus* under salt stress. Ind. Crop Prod..

[17] Tlahig S., Bellani L., Karmous I., Barbieri F., Loumerem M., Muccifora S. (2021). Response to salinity in legume species: An insight on the effects of salt stress during seed germination and seedling growth. Chem. Biodivers..

[18] Zhang Q., Zheng G., Wang Q., Zhu J., Zhou Z., Zhou W., Xu J., Sun H., Zhong J., Gu Y. (2022). Molecular mechanisms of flavonoid accumulation in germinating common bean (*Phaseolus vulgaris*) under salt stress. Front. Nutr..

[19] Xu N., Liu S., Lu Z., Pang S., Wang L., Wang L., Li W. (2020). Gene expression profiles and flavonoid accumulation during salt stress in *Ginkgo biloba* seedlings. Plants.

[20] Zhao X., Wu T., Guo S., Hu J., Zhan Y. (2022). Ectopic expression of AeNAC83, a NAC Transcription factor from *Abelmoschus esculentus*, inhibits growth and confers tolerance to salt stress in *Arabidopsis*. Int. J. Mol. Sci..

[21] Zhang X., Shen Y., Mu K., Cai W., Zhao Y., Shen H., Wang X., Ma H. (2022). Phenylalanine ammonia lyase *GmPAL1.1* promotes seed vigor under high-temperature and -humidity stress and enhances seed germination under salt and drought stress in transgenic *Arabidopsis*. Plants.

[22] Luo J., Butelli E., Hill L., Parr A., Niggeweg R., Bailey P., Weisshaar B., Martin C. (2008). AtMYB12 regulates caffeoyl quinic acid and flavonol synthesis in tomato: Expression in fruit results in very high levels of both types of polyphenol. Plant J..

[23] Hussain S., Morillon R., Anjum M.A., Ollitrault P., Costantino G., Luro F. (2014). Genetic diversity revealed by physiological behavior of citrus genotypes subjected to salt stress. Acta Physiol. Plant.

[24] Lu Q., Jin L., Wang P., Liu F., Huang B., Wen M., Wu S. (2023). Effects of interaction of protein hydrolysate and arbuscular mycorrhizal fungi effects on citrus growth and expressions of stress-responsive genes (aquaporins and SOSs) under salt stress. J. Fungi.

[25] Danquah A., de Zelicourt A., Colcombet J., Hirt H. (2014). The role of ABA and MAPK signaling pathways in plant abiotic stress responses. Biotechnol. Adv..

[26] Kong W., Zhong H., Gong Z., Fang X., Sun T., Deng X., Li Y. (2019). Meta-analysis of salt stress transcriptome responses in different rice genotypes at the seedling stage. Plants.

[27] Duan S., Xu Z., Li X., Liao P., Qin H., Mao Y., Dai W., Ma H., Bao M. (2022). Dodder-transmitted mobile systemic signals activate a salt-stress response characterized by a transcriptome change in *Citrus sinensis*. Front. Plant Sci..

[28] Xu J., Li Y., Wang Y., Liu H., Lei L., Yang H., Liu G., Ren D. (2008). Activation of MAPK kinase 9 induces ethylene and camalexin biosynthesis and enhances sensitivity to salt stress in *Arabidopsis*. J. Biol. Chem..

[29] Conroy C., Ching J., Gao Y., Wang X., Rampitsch C., Xing T. (2013). Knockout of *AtMKK1* enhances salt tolerance and modifies metabolic activities in *Arabidopsis*. Plant Signal Behav..

[30] Shen N., Wang T., Gan Q., Liu S., Wang L., Jin B. (2022). Plant flavonoids: Classification, distribution, biosynthesis, and antioxidant activity. Food Chem..

[31] Agati G., Biricolti S., Guidi L., Ferrini F., Fini A., Tattini M. (2011). The biosynthesis of flavonoids is enhanced similarly by UV radiation and root zone salinity in L. vulgare leaves. J. Plant Physiol..

[32] Ren G., Yang P., Cui J., Gao Y., Yin C., Bai Y., Zhao D., Chang J. (2022). Multiomics analyses of two sorghum cultivars reveal the molecular mechanism of salt tolerance. Front. Plant Sci..

[33] Akhtar M.T., Noor M., Lin X., Lu Z., Jin B. (2026). Flavonoids in plant salt stress responses: Biosynthesis, regulation, functions, and signaling networks. Plants.

[34] Wang Y., Liu X., Chen J., Cao J., Li X., Sun C. (2022). Citrus flavonoids and their antioxidant evaluation. Crit. Rev. Food Sci..

[35] Shah A., Smith D.L. (2020). Flavonoids in agriculture: Chemistry and roles in, biotic and abiotic stress responses, and microbial associations. Agronomy.

[36] Commisso M., Toffali K., Strazzer P., Stocchero M., Ceoldo S., Baldan B., Levi M., Guzzo F. (2016). Impact of phenylpropanoid compounds on heat stress tolerance in carrot cell cultures. Front. Plant Sci..

[37] Mierziak J., Kostyn K., Kulma A. (2014). Flavonoids as important molecules of plant interactions with the environment. Molecules.

[38] Ithal N., Reddy A.R. (2004). Rice flavonoid pathway genes, OsDfr and OsAns, are induced by dehydration, high salt and ABA, and contain stress responsive promoter elements that interact with the transcription activator, OsC1-MYB. Plant Sci..

[39] Parvin K., Hasanuzzaman M., Bhuyan M., Mohsin S.M., Fujita A.M. (2019). Quercetin mediated salt tolerance in tomato through the enhancement of plant antioxidant defense and glyoxalase systems. Plants.

[40] Rao M.J., Duan M., Eman M., Yuan H., Sharma A., Zheng B. (2024). Comparative analysis of citrus species’ flavonoid metabolism, gene expression profiling, and their antioxidant capacity under drought stress. Antioxid..

[41] Wan H., Liu Y., Wang T., Jiang P., Wen W., Nie J. (2023). Combined transcriptomic and metabolomic analyses identifies CsERF003, a citrus ERF transcription factor, as flavonoid activator. Plant Sci..

[42] Hassani D., Fu X., Shen Q., Khalid M., Rose J.K.C., Tang K. (2020). Parallel Transcriptional regulation of artemisinin and flavonoid biosynthesis. Trends Plant Sci..

[43] Grunewald W., De Smet I., Lewis D.R., Löfke C., Jansen L., Goeminne G., Vanden Bossche R., Karimi M., De Rybel B., Vanholme B. (2012). Transcription factor WRKY23 assists auxin distribution patterns during *Arabidopsis* root development through local control on flavonol biosynthesis. Proc. Natl. Acad. Sci. USA.

[44] Li C., Yu W., Xu J., Lu X., Liu Y. (2022). Anthocyanin biosynthesis induced by myb transcription factors in plants. Int. J. Mol. Sci..

[45] Xu W., Dubos C., Lepiniec L. (2015). Transcriptional control of flavonoid biosynthesis by MYB-bHLH-WDR complexes. Trends Plant Sci..

[46] An X., Tian Y., Chen K., Liu X., Liu D., Xie X., Cheng C., Cong P., Hao Y. (2015). MdMYB9 and MdMYB11 are involved in the regulation of the JA-induced biosynthesis of anthocyanin and proanthocyanidin in apples. Plant Cell Physiol..

[47] Huang Y., Xu Y., Jiang X., Yu H., Jia H., Tan C., Hu G., Hu Y., Rao M.J., Deng X. (2021). Genome of a citrus rootstock and global DNA demethylation caused by heterografting. Hortic. Res..

