# Supplementary figures and images for: Integrative Analysis of Transcriptome and Metabolome Reveals Molecular Mechanisms of Salt Tolerance in Two Citrus Rootstocks
Source: Int J Mol Sci. 2026 Jun 14;27(12):5361. doi: 10.3390/ijms27125361 (PMC13299380; doi:10.3390/ijms27125361)

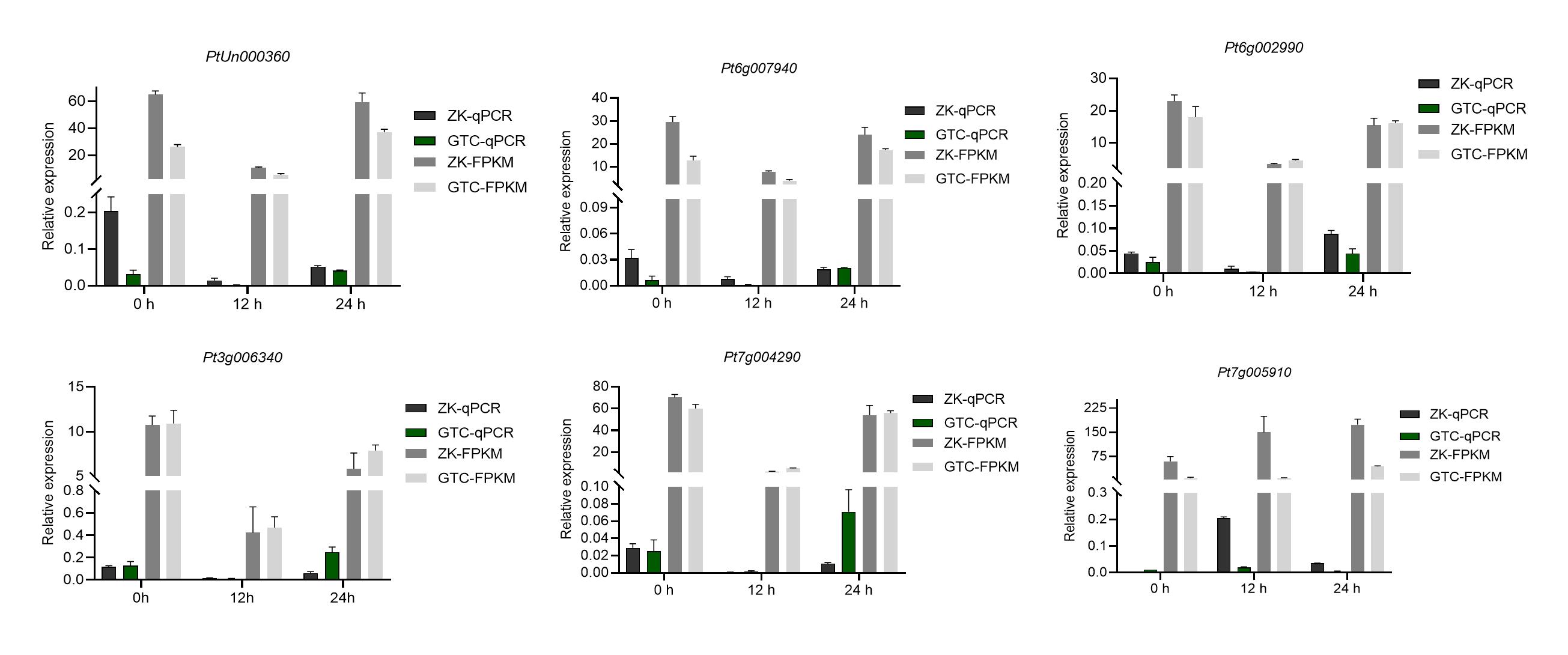

Supplement: Supplementary file 1 [file ijms-27-05361-s001.zip › Figure S1 Validation of RNA-seq data using RT-qPCR..jpg]
